# Supplementary figures and images for: Accessible molecular phylogenomics at no cost: obtaining 14 new mitogenomes for the ant subfamily Pseudomyrmecinae from public data
Source: PeerJ. 2019 Jan 24;7:e6271. doi: 10.7717/peerj.6271 (PMC6348091; doi:10.7717/peerj.6271)

A)

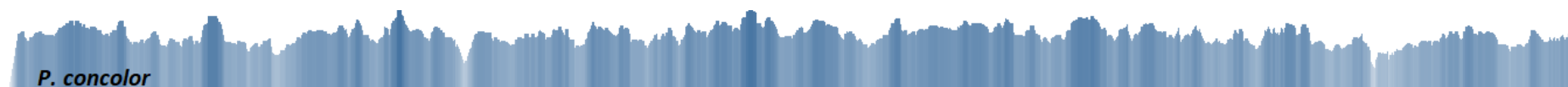

B)

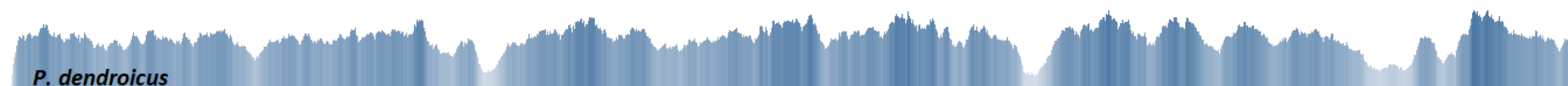

C)

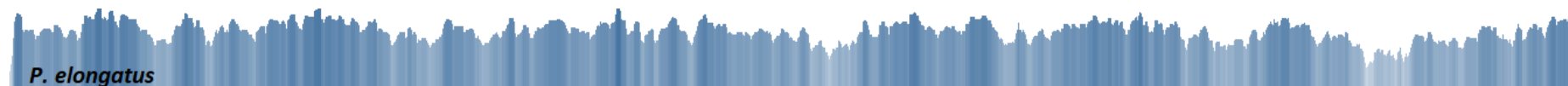

D)

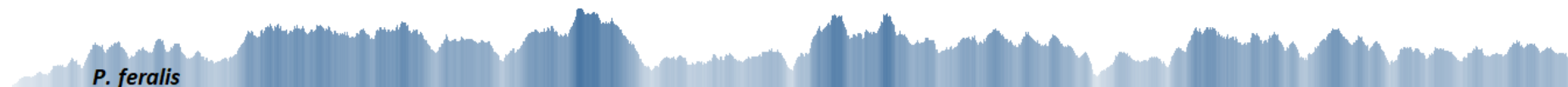

E)

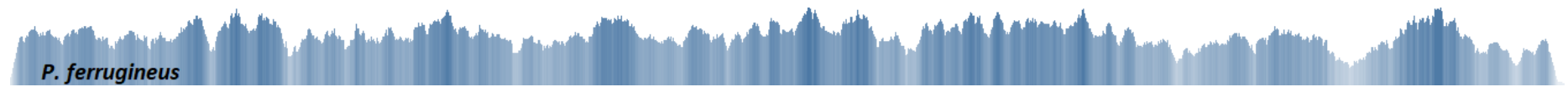

F)

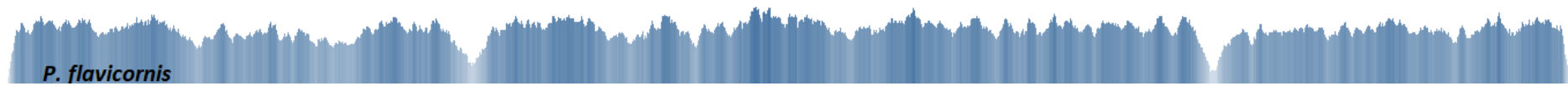

G)

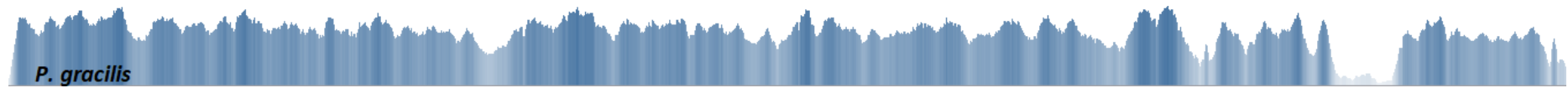

H)

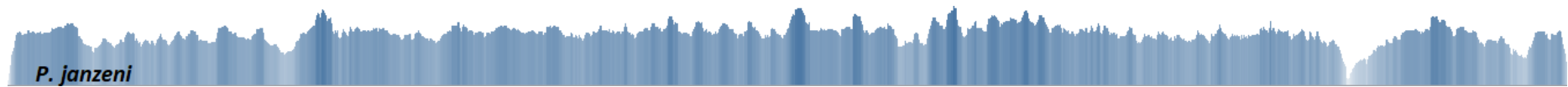

I)

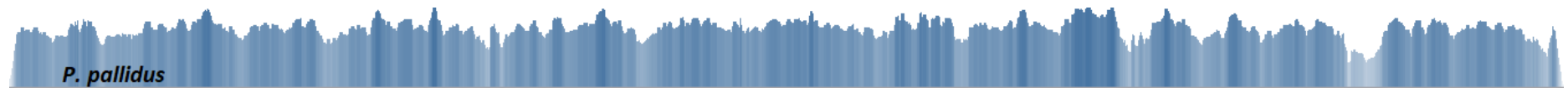

J)

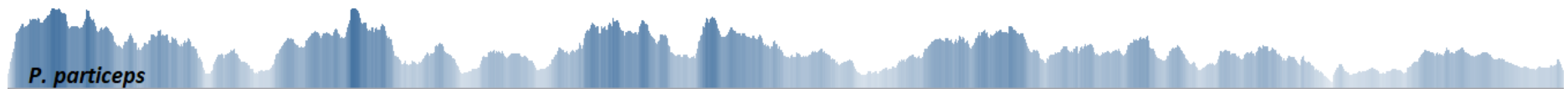

K)

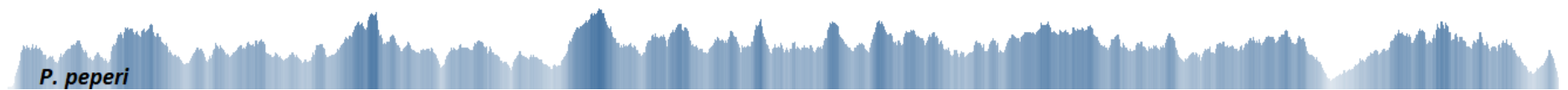

L)

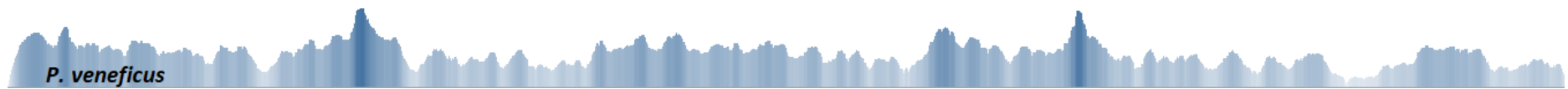

M)

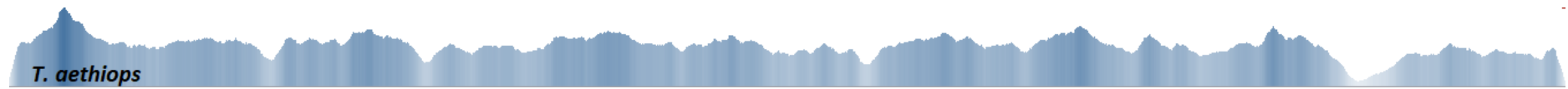

N)

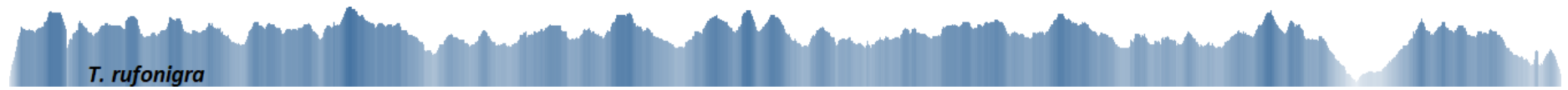

Supplement: Figure S1 — The species are presented in the following order: (A) P. concolor. (B) P. dendroicus. (C) P. elongatus. (D) P. feralis. (E) P.ferrugineus. (F) P. flavicornis. (G) P. gracilis. (H) P. janzeni. (I) P. pallidus. (J) P. particeps. (K) P. peperi. (L) P. veneficus. (M) T. aethiops. (N) T. rufonigra. No high-coverage peaks are obseverd in any species and low-coverage regions are present in G, H, J, K, L, M and N. [file peerj-07-6271-s001.pdf]
