# Supplementary material for: Accessible molecular phylogenomics at no cost: obtaining 14 new mitogenomes for the ant subfamily Pseudomyrmecinae from public data
Source: PeerJ. 2019 Jan 24;7:e6271. doi: 10.7717/peerj.6271 (PMC6348091; doi:10.7717/peerj.6271)
Supplement: Table S1 [file peerj-07-6271-s003.docx]

| **Species name** | **Bioproject** | **Experiment** | **Biosample** | **SRA Run number** | **FTP link used for dataset download** | **Dataset type** | **# Downloaded Sequencing Reads** | **#Bases** | **Reference** |
| --- | --- | --- | --- | --- | --- | --- | --- | --- | --- |
| *Pseudomyrmex concolor* | PRJNA268384 | SRX831102 | SAMN03275516 | SRR1742927 | ftp://ftp.sra.ebi.ac.uk/vol1/srr/SRR174/007/SRR1742927 | WGS | 359,475,424 | 35.9 Gbp | Rubin & Moreau, 2016 |
| *Pseudomyrmex dendroicus* | PRJNA268384 | SRX831097 | SAMN03275515 | SRR1742922 | ftp://ftp.sra.ebi.ac.uk/vol1/srr/SRR174/002/SRR1742922 | WGS | 366,341,280 | 36.6 Gbp | Rubin & Moreau, 2016 |
| *Pseudomyrmex elongatus* | PRJNA268384 | SRX831106 | SAMN03275518 | SRR1742975 | ftp://ftp.sra.ebi.ac.uk/vol1/srr/SRR174/005/SRR1742975 | WGS | 409,687,406 | 41 Gbp | Rubin & Moreau, 2016 |
| *Pseudomyrmex feralis* | PRJNA357470 | SRX2424867 | SAMN06141944 | SRR5112519 | ftp://ftp.sra.ebi.ac.uk/vol1/srr/SRR511/009/SRR5112519 | UCE | 4,552,328 | 569 Mbp | Ward & Branstetter, 2017 |
| *Pseudomyrmex ferrugineus* | PRJNA357470 | SRX2424886 | SAMN06141956 | SRR5112538 | ftp://ftp.sra.ebi.ac.uk/vol1/srr/SRR511/008/SRR5112538 | UCE | 5,274,142 | 659.3 Mbp | Ward & Branstetter, 2017 |
| *Pseudomyrmex flavicornis* | PRJNA268384 | SRX831107 | SAMN03275519 | SRR1742976 | ftp://ftp.sra.ebi.ac.uk/vol1/srr/SRR174/006/SRR1742976 | WGS | 290,503,558 | 29.1 Gbp | Rubin & Moreau, 2016 |
| *Pseudomyrmex gracilis* | PRJNA268384 | SRX831110 | SAMN03219222 | SRR1742979 | ftp://ftp.sra.ebi.ac.uk/vol1/srr/SRR174/009/SRR1742979 | WGS | 358,526,654 | 35.9 Gbp | Rubin & Moreau, 2016 |
| *Pseudomyrmex janzeni* | PRJNA357470 | SRX2424860 | SAMN06141954 | SRR5112512 | ftp://ftp.sra.ebi.ac.uk/vol1/srr/SRR511/002/SRR5112512 | UCE | 3,720,456 | 465.1 Mbp | Ward & Branstetter, 2017 |
| *Pseudomyrmex pallidus* | PRJNA268384 | SRX831105 | SAMN03275517 | SRR1742947 | ftp://ftp.sra.ebi.ac.uk/vol1/srr/SRR174/007/SRR1742947 | WGS | 342,184,040 | 34.2 Gbp | Rubin & Moreau, 2016 |
| *Pseudomyrmex particeps* | PRJNA357470 | SRX2424875 | SAMN06141966 | SRR5112527 | ftp://ftp.sra.ebi.ac.uk/vol1/srr/SRR511/007/SRR5112527 | UCE | 7,821,658 | 977.7 Mbp | Ward & Branstetter, 2017 |
| *Pseudomyrmex peperi* | PRJNA357470 | SRX2424871 | SAMN06141946 | SRR5112523 | ftp://ftp.sra.ebi.ac.uk/vol1/srr/SRR511/003/SRR5112523 | UCE | 4,383,700 | 548 Mbp | Ward & Branstetter, 2017 |
| *Pseudomyrmex veneficus* | PRJNA357470 | SRX2424879 | SAMN06141958 | SRR5112531 | ftp://ftp.sra.ebi.ac.uk/vol1/srr/SRR511/001/SRR5112531 | UCE | 9,267,862 | 587.6 Mbp | Ward & Branstetter, 2017 |
| *Tetraponera aethiops* | PRJNA360290 | SRX2468757 | SAMN06208957 | SRR5150667 | ftp://ftp.sra.ebi.ac.uk/vol1/srr/SRR515/007/SRR5150667 | UCE | 10,915,176 | 1.4 Gbp | Branstetter et al., 2017 |
| *Tetraponera rufonigra* | PRJNA293213 | SRX1164924 | SAMN03998752 | SRR2184195 | ftp://ftp.sra.ebi.ac.uk/vol1/srr/SRR218/005/SRR2184195 | UCE | 5,250,766 | 733.3 Mbp | N/A |
