# Supplementary material for: Accessible molecular phylogenomics at no cost: obtaining 14 new mitogenomes for the ant subfamily Pseudomyrmecinae from public data
Source: PeerJ. 2019 Jan 24;7:e6271. doi: 10.7717/peerj.6271 (PMC6348091; doi:10.7717/peerj.6271)
Supplement: Table S2 [file peerj-07-6271-s004.docx]

| 1 |  | |  | |  | | ***Pseudomyrmex gracilis*** | | | |  | | |  | | | | |  | | | | |  | | | | | | | |
| --- | --- | --- | --- | --- | --- | --- | --- | --- | --- | --- | --- | --- | --- | --- | --- | --- | --- | --- | --- | --- | --- | --- | --- | --- | --- | --- | --- | --- | --- | --- | --- |
| **Gene** | **Position** | | | | **Size** | | | | | | **Codon** | | | | | | | | **Intergenic** | | | | |  | | | | | | | |
|  | **From** | | **To** | | **Nucleotide** | | **Aminoacid** | | | | **Start** | | | **Stop** | | | | | **nucleotide** | | | | |  | | | | | | | |
| COX1 | 1 | | 1533 | | 1533 | | 510 | | | | ATG | | | TAA | | | | | 0 | | | | | + | | | | | | | |
| tRNA(Leu) | 1534 | | 1599 | | 66 | |  | | | |  | | |  | | | | | 0 | | | | | + | | | | | | | |
| COX2 | 1600 | | 2271 | | 672 | | 223 | | | | ATC | | | TAA | | | | | 16 | | | | | + | | | | | | | |
| tRNA(Lys) | 2288 | | 2359 | | 72 | |  | | | |  | | |  | | | | | 0 | | | | | + | | | | | | | |
| tRNA(Asp) | 2360 | | 2422 | | 63 | |  | | | |  | | |  | | | | | 0 | | | | | + | | | | | | | |
| ATP8 | 2423 | | 2584 | | 162 | | 53 | | | | ATT | | | TAA | | | | | 21 | | | | | + | | | | | | | |
| ATP6 | 2606 | | 3272 | | 667 | | 222 | | | | ATG | | | T- | | | | | 0 | | | | | + | | | | | | | |
| COX3 | 3273 | | 4052 | | 780 | | 259 | | | | ATG | | | TAA | | | | | 3 | | | | | + | | | | | | | |
| tRNA(Gly) | 4056 | | 4125 | | 70 | |  | | | |  | | |  | | | | | 3 | | | | | + | | | | | | | |
| ND3 | 4129 | | 4479 | | 351 | | 116 | | | | ATG | | | TAA | | | | | 6 | | | | | + | | | | | | | |
| tRNA(Ala) | 4486 | | 4547 | | 62 | |  | | | |  | | |  | | | | | 0 | | | | | + | | | | | | | |
| tRNA(Arg) | 4548 | | 4615 | | 68 | |  | | | |  | | |  | | | | | 2 | | | | | + | | | | | | | |
| tRNA(Asn) | 4618 | | 4684 | | 67 | |  | | | |  | | |  | | | | | 28 | | | | | + | | | | | | | |
| tRNA(Ser) | 4713 | | 4772 | | 60 | |  | | | |  | | |  | | | | | 5 | | | | | + | | | | | | | |
| tRNA(Glu) | 4778 | | 4850 | | 73 | |  | | | |  | | |  | | | | | 10 | | | | | + | | | | | | | |
| tRNA(Phe) | 4861 | | 4929 | | 69 | |  | | | |  | | |  | | | | | 39 | | | | | - | | | | | | | |
| ND5 | 4969 | | 6633 | | 1665 | | 554 | | | | ATT | | | TAA | | | | | 0 | | | | | - | | | | | | | |
| tRNA(His) | 6634 | | 6698 | | 65 | |  | | | |  | | |  | | | | | 0 | | | | | - | | | | | | | |
| ND4 | 6699 | | 8022 | | 1324 | | 441 | | | | ATG | | | T- | | | | | 0 | | | | | - | | | | | | | |
| ND4L | 8023 | | 8305 | | 283 | | 94 | | | | ATA | | | T- | | | | | 27 | | | | | - | | | | | | | |
| tRNA(Thr) | 8333 | | 8398 | | 66 | |  | | | |  | | |  | | | | | 1 | | | | | + | | | | | | | |
| tRNA(Pro) | 8400 | | 8466 | | 67 | |  | | | |  | | |  | | | | | 20 | | | | | - | | | | | | | |
| ND6 | 8487 | | 9018 | | 532 | | 177 | | | | ATG | | | T- | | | | | 0 | | | | | + | | | | | | | |
| CYTB | 9019 | | 10139 | | 1120 | | 373 | | | | ATG | | | TA- | | | | | 0 | | | | | + | | | | | | | |
| tRNA(Ser) | 10140 | | 10208 | | 69 | |  | | | |  | | |  | | | | | 11 | | | | | + | | | | | | | |
| ND1 | 10220 | | 11170 | | 951 | | 316 | | | | ATT | | | TAA | | | | | 0 | | | | | - | | | | | | | |
| tRNA(Leu) | 11171 | | 11238 | | 68 | |  | | | |  | | |  | | | | | 11 | | | | | - | | | | | | | |
| 16S rRNA | 11250 | | 12527 | | 1278 | |  | | | |  | | |  | | | | | 20 | | | | | - | | | | | | | |
| tRNA(Val) | 12548 | | 12615 | | 68 | |  | | | |  | | |  | | | | | 10 | | | | | - | | | | | | | |
| 12S rRNA | 12626 | | 13401 | | 776 | |  | | | |  | | |  | | | | | 628 | | | | | - | | | | | | | |
| tRNA(Met) | 14030 | | 14098 | | 69 | |  | | | |  | | |  | | | | | 0 | | | | | + | | | | | | | |
| tRNA(Ile) | 14099 | | 14165 | | 67 | |  | | | |  | | |  | | | | | 45 | | | | | + | | | | | | | |
| tRNA(Gln) | 14211 | | 14280 | | 70 | |  | | | |  | | |  | | | | | 61 | | | | | - | | | | | | | |
| ND2 | 14342 | | 15303 | | 961 | | 320 | | | | ATT | | | TA- | | | | | 0 | | | | | + | | | | | | | |
| tRNA(Trp) | 15304 | | 15369 | | 66 | |  | | | |  | | |  | | | | | 1 | | | | | + | | | | | | | |
| tRNA(Cys) | 15371 | | 15434 | | 64 | |  | | | |  | | |  | | | | | 38 | | | | | - | | | | | | | |
| tRNA(Tyr) | 15473 | | 15538 | | 66 | |  | | | |  | | |  | | | | | 166 | | | | | - | | | | | | | |
| 2 |  | |  | | ***Pseudomyrmex  concolor*** | | | | | | | | |  | | | | |  | | | | |  | | | | | | | |
| **Gene** | **Position** | | | | **Size** | | | | | | **Codon** | | | | | | | | **Intergenic** | | | | |  | | | | | | | |
|  | **From** | | **To** | | **Nucleotide** | | **Aminoacid** | | | | **Start** | | | **Stop** | | | | | **nucleotide** | | | | |  | | | | | | | |
| COX1 | 1 | | 1533 | | 1533 | | 510 | | | | ATG | | | TAA | | | | | 2 | | | | | + | | | | | | | |
| tRNA(Leu) | 1536 | | 1607 | | 72 | |  | | | |  | | |  | | | | | 0 | | | | | + | | | | | | | |
| COX2 | 1608 | | 2285 | | 678 | | 225 | | | | ATA | | | TAA | | | | | 60 | | | | | + | | | | | | | |
| tRNA(Lys) | 2346 | | 2415 | | 70 | |  | | | |  | | |  | | | | | 0 | | | | | + | | | | | | | |
| tRNA(Asp) | 2416 | | 2482 | | 67 | |  | | | |  | | |  | | | | | 0 | | | | | + | | | | | | | |
| ATP8 | 2483 | | 2662 | | 180 | | 59 | | | | ATT | | | TAA | | | | | 35 | | | | | + | | | | | | | |
| ATP6 | 2698 | | 3361 | | 664 | | 221 | | | | ATG | | | T- | | | | | 0 | | | | | + | | | | | | | |
| COX3 | 3362 | | 4141 | | 780 | | 259 | | | | ATG | | | TAA | | | | | 26 | | | | | + | | | | | | | |
| tRNA(Gly) | 4168 | | 4245 | | 78 | |  | | | |  | | |  | | | | | 4 | | | | | + | | | | | | | |
| ND3 | 4250 | | 4597 | | 348 | | 115 | | | | ATG | | | TAA | | | | | 71 | | | | | + | | | | | | | |
| tRNA(Ala) | 4669 | | 4737 | | 69 | |  | | | |  | | |  | | | | | 0 | | | | | + | | | | | | | |
| tRNA(Arg) | 4738 | | 4805 | | 68 | |  | | | |  | | |  | | | | | 7 | | | | | + | | | | | | | |
| tRNA(Asn) | 4813 | | 4879 | | 67 | |  | | | |  | | |  | | | | | 15 | | | | | + | | | | | | | |
| tRNA(Ser) | 4895 | | 4952 | | 58 | |  | | | |  | | |  | | | | | 19 | | | | | + | | | | | | | |
| tRNA(Glu) | 4972 | | 5044 | | 73 | |  | | | |  | | |  | | | | | 106 | | | | | + | | | | | | | |
| tRNA(Phe) | 5151 | | 5220 | | 70 | |  | | | |  | | |  | | | | | 3 | | | | | - | | | | | | | |
| ND5 | 5224 | | 6885 | | 1662 | | 553 | | | | GTG | | | TAA | | | | | 0 | | | | | - | | | | | | | |
| tRNA(His) | 6886 | | 6954 | | 69 | |  | | | |  | | |  | | | | | 6 | | | | | - | | | | | | | |
| ND4 | 6961 | | 8283 | | 1323 | | 440 | | | | ATG | | | TAA | | | | | 0 | | | | | - | | | | | | | |
| ND4L | 8284 | | 8566 | | 283 | | 94 | | | | ATA | | | T- | | | | | 97 | | | | | - | | | | | | | |
| tRNA(Thr) | 8664 | | 8730 | | 67 | |  | | | |  | | |  | | | | | 26 | | | | | + | | | | | | | |
| tRNA(Pro) | 8757 | | 8823 | | 67 | |  | | | |  | | |  | | | | | 17 | | | | | - | | | | | | | |
| ND6 | 8841 | | 9372 | | 532 | | 177 | | | | ATG | | | T- | | | | | 0 | | | | | + | | | | | | | |
| CYTB | 9373 | | 10493 | | 1121 | | 373 | | | | ATG | | | TA- | | | | | 0 | | | | | + | | | | | | | |
| tRNA(Ser) | 10494 | | 10563 | | 70 | |  | | | |  | | |  | | | | | 3 | | | | | + | | | | | | | |
| ND1 | 10567 | | 11514 | | 948 | | 315 | | | | ATT | | | TAG | | | | | 0 | | | | | - | | | | | | | |
| tRNA(Leu) | 11515 | | 11582 | | 68 | |  | | | |  | | |  | | | | | 7 | | | | | - | | | | | | | |
| 16S rRNA | 11590 | | 12880 | | 1291 | |  | | | |  | | |  | | | | | 0 | | | | | - | | | | | | | |
| tRNA(Val) | 12881 | | 12971 | | 91 | |  | | | |  | | |  | | | | | 2 | | | | | - | | | | | | | |
| 12S rRNA | 12974 | | 13760 | | 787 | |  | | | |  | | |  | | | | | 527 | | | | | - | | | | | | | |
| tRNA(Met) | 14288 | | 14357 | | 70 | |  | | | |  | | |  | | | | | 5 | | | | | + | | | | | | | |
| tRNA(Ile) | 14363 | | 14431 | | 69 | |  | | | |  | | |  | | | | | 40 | | | | | + | | | | | | | |
| tRNA(Gln) | 14472 | | 14546 | | 75 | |  | | | |  | | |  | | | | | 56 | | | | | - | | | | | | | |
| ND2 | 14603 | | 15571 | | 969 | | 322 | | | | ATA | | | TAA | | | | | 8 | | | | | + | | | | | | | |
| tRNA(Trp) | 15580 | | 15650 | | 71 | |  | | | |  | | |  | | | | | 20 | | | | | + | | | | | | | |
| tRNA(Cys) | 15671 | | 15733 | | 63 | |  | | | |  | | |  | | | | | 48 | | | | | - | | | | | | | |
| tRNA(Tyr) | 15782 | | 15850 | | 69 | |  | | | |  | | |  | | | | | 56 | | | | | - | | | | | | | |
| 3 |  | |  | | ***Pseudomyrmex  elongatus*** | | | | | | | | |  | | | | |  | | | | |  | | | | | | | |
| **Gene** | **Position** | | | | **Size** | | | | | | **Codon** | | | | | | | | **Intergenic** | | | | |  | | | | | | | |
|  | **From** | | **To** | | **Nucleotide** | | **Aminoacid** | | | | **Start** | | | **Stop** | | | | | **nucleotide** | | | | |  | | | | | | | |
| COX1 | 1 | | 1531 | | 1531 | | 510 | | | | ATG | | | T- | | | | | 0 | | | | | + | | | | | | | |
| tRNA(Leu) | 1532 | | 1599 | | 68 | |  | | | |  | | |  | | | | | 0 | | | | | + | | | | | | | |
| COX2 | 1600 | | 2277 | | 678 | | 225 | | | | ATT | | | TAA | | | | | 94 | | | | | + | | | | | | | |
| tRNA(Lys) | 2372 | | 2444 | | 73 | |  | | | |  | | |  | | | | | 0 | | | | | + | | | | | | | |
| tRNA(Asp) | 2445 | | 2513 | | 69 | |  | | | |  | | |  | | | | | 0 | | | | | + | | | | | | | |
| ATP8 | 2514 | | 2690 | | 177 | | 58 | | | | ATT | | | TAA | | | | | 165 | | | | | + | | | | | | | |
| ATP6 | 2856 | | 3522 | | 667 | | 222 | | | | ATG | | | T- | | | | | 0 | | | | | + | | | | | | | |
| COX3 | 3523 | | 4302 | | 780 | | 259 | | | | ATG | | | TAA | | | | | 15 | | | | | + | | | | | | | |
| tRNA(Gly) | 4318 | | 4385 | | 68 | |  | | | |  | | |  | | | | | 5 | | | | | + | | | | | | | |
| ND3 | 4391 | | 4738 | | 348 | | 115 | | | | ATG | | | TAA | | | | | 62 | | | | | + | | | | | | | |
| tRNA(Ala) | 4801 | | 4869 | | 69 | |  | | | |  | | |  | | | | | 0 | | | | | + | | | | | | | |
| tRNA(Arg) | 4870 | | 4937 | | 68 | |  | | | |  | | |  | | | | | 0 | | | | | + | | | | | | | |
| tRNA(Asn) | 4938 | | 5008 | | 71 | |  | | | |  | | |  | | | | | 9 | | | | | + | | | | | | | |
| tRNA(Ser) | 5018 | | 5079 | | 62 | |  | | | |  | | |  | | | | | 35 | | | | | + | | | | | | | |
| tRNA(Glu) | 5115 | | 5184 | | 70 | |  | | | |  | | |  | | | | | 159 | | | | | + | | | | | | | |
| tRNA(Phe) | 5344 | | 5412 | | 69 | |  | | | |  | | |  | | | | | 12 | | | | | - | | | | | | | |
| ND5 | 5425 | | 7092 | | 1668 | | 555 | | | | ATG | | | TAG | | | | | 0 | | | | | - | | | | | | | |
| tRNA(His) | 7093 | | 7159 | | 67 | |  | | | |  | | |  | | | | | 12 | | | | | - | | | | | | | |
| ND4 | 7172 | | 8503 | | 1332 | | 443 | | | | ATG | | | TAA | | | | | 0 | | | | | - | | | | | | | |
| ND4L | 8504 | | 8786 | | 283 | | 94 | | | | ATA | | | T- | | | | | 129 | | | | | - | | | | | | | |
| tRNA(Thr) | 8916 | | 8984 | | 69 | |  | | | |  | | |  | | | | | 131 | | | | | + | | | | | | | |
| tRNA(Pro) | 9116 | | 9180 | | 65 | |  | | | |  | | |  | | | | | 136 | | | | | - | | | | | | | |
| ND6 | 9317 | | 9848 | | 532 | | 177 | | | | ATG | | | T- | | | | | 0 | | | | | + | | | | | | | |
| CYTB | 9849 | | 10964 | | 1116 | | 371 | | | | ATG | | | TAG | | | | | 14 | | | | | + | | | | | | | |
| tRNA(Ser) | 10979 | | 11050 | | 72 | |  | | | |  | | |  | | | | | 604 | | | | | + | | | | | | | |
| ND1 | 11655 | | 12605 | | 951 | | 316 | | | | ATG | | | TAA | | | | | 0 | | | | | - | | | | | | | |
| tRNA(Leu) | 12606 | | 12672 | | 67 | |  | | | |  | | |  | | | | | 9 | | | | | - | | | | | | | |
| 16S rRNA | 12682 | | 14008 | | 1327 | |  | | | |  | | |  | | | | | 34 | | | | | - | | | | | | | |
| tRNA(Val) | 14043 | | 14097 | | 55 | |  | | | |  | | |  | | | | | 23 | | | | | - | | | | | | | |
| 12S rRNA | 14121 | | 14906 | | 786 | |  | | | |  | | |  | | | | | 697 | | | | | - | | | | | | | |
| tRNA(Met) | 15604 | | 15673 | | 70 | |  | | | |  | | |  | | | | | 6 | | | | | + | | | | | | | |
| tRNA(Ile) | 15680 | | 15747 | | 68 | |  | | | |  | | |  | | | | | 37 | | | | | + | | | | | | | |
| tRNA(Gln) | 15785 | | 15854 | | 70 | |  | | | |  | | |  | | | | | 72 | | | | | - | | | | | | | |
| ND2 | 15927 | | 16892 | | 966 | | 321 | | | | ATA | | | TAA | | | | | 2 | | | | | + | | | | | | | |
| tRNA(Trp) | 16895 | | 16964 | | 70 | |  | | | |  | | |  | | | | | 40 | | | | | + | | | | | | | |
| tRNA(Cys) | 17005 | | 17075 | | 71 | |  | | | |  | | |  | | | | | 92 | | | | | - | | | | | | | |
| tRNA(Tyr) | 17168 | | 17236 | | 69 | |  | | | |  | | |  | | | | | 68 | | | | | - | | | | | | | |
| 4 |  | |  | | ***Pseudomyrmex dendroicus*** | | | | | | | | |  | | | | |  | | | | |  | | | | | | | |
| **Gene** | **Position** | | | | **Size** | | | | | | **Codon** | | | | | | | | **Intergenic** | | | | |  | | | | | | | |
|  | **From** | | **To** | | **Nucleotide** | | **Aminoacid** | | | | **Start** | | | **Stop** | | | | | **nucleotide** | | | | |  | | | | | | | |
| COX1 | 1 | | 1533 | | 1533 | | 510 | | | | ATG | | | TAA | | | | | 6 | | | | | + | | | | | | | |
| tRNA(Leu) | 1540 | | 1608 | | 69 | |  | | | |  | | |  | | | | | 0 | | | | | + | | | | | | | |
| COX2 | 1609 | | 2286 | | 678 | | 225 | | | | ATT | | | TAA | | | | | 88 | | | | | + | | | | | | | |
| tRNA(Lys) | 2375 | | 2446 | | 72 | |  | | | |  | | |  | | | | | 0 | | | | | + | | | | | | | |
| tRNA(Asp) | 2447 | | 2512 | | 66 | |  | | | |  | | |  | | | | | 0 | | | | | + | | | | | | | |
| ATP8 | 2513 | | 2686 | | 174 | | 57 | | | | ATT | | | TAA | | | | | 147 | | | | | + | | | | | | | |
| ATP6 | 2834 | | 3500 | | 667 | | 222 | | | | ATG | | | T- | | | | | 0 | | | | | + | | | | | | | |
| COX3 | 3501 | | 4280 | | 780 | | 259 | | | | ATG | | | TAA | | | | | 7 | | | | | + | | | | | | | |
| tRNA(Gly) | 4288 | | 4359 | | 72 | |  | | | |  | | |  | | | | | 2 | | | | | + | | | | | | | |
| ND3 | 4362 | | 4709 | | 348 | | 115 | | | | ATG | | | TAA | | | | | 71 | | | | | + | | | | | | | |
| tRNA(Ala) | 4781 | | 4848 | | 68 | |  | | | |  | | |  | | | | | 0 | | | | | + | | | | | | | |
| tRNA(Arg) | 4849 | | 4919 | | 71 | |  | | | |  | | |  | | | | | 6 | | | | | + | | | | | | | |
| tRNA(Asn) | 4926 | | 4994 | | 69 | |  | | | |  | | |  | | | | | 10 | | | | | + | | | | | | | |
| tRNA(Ser) | 5005 | | 5068 | | 64 | |  | | | |  | | |  | | | | | 40 | | | | | + | | | | | | | |
| tRNA(Glu) | 5109 | | 5178 | | 70 | |  | | | |  | | |  | | | | | 266 | | | | | + | | | | | | | |
| tRNA(Phe) | 5445 | | 5514 | | 70 | |  | | | |  | | |  | | | | | 10 | | | | | - | | | | | | | |
| ND5 | 5525 | | 7192 | | 1668 | | 555 | | | | ATA | | | TAA | | | | | 0 | | | | | - | | | | | | | |
| tRNA(His) | 7193 | | 7262 | | 70 | |  | | | |  | | |  | | | | | 25 | | | | | - | | | | | | | |
| ND4 | 7288 | | 8625 | | 1338 | | 445 | | | | ATG | | | TAA | | | | | 2 | | | | | - | | | | | | | |
| ND4L | 8628 | | 8908 | | 281 | | 94 | | | | ATT | | | T- | | | | | 125 | | | | | - | | | | | | | |
| tRNA(Thr) | 9034 | | 9098 | | 65 | |  | | | |  | | |  | | | | | 112 | | | | | + | | | | | | | |
| tRNA(Pro) | 9211 | | 9278 | | 68 | |  | | | |  | | |  | | | | | 52 | | | | | - | | | | | | | |
| ND6 | 9331 | | 9862 | | 532 | | 177 | | | | ATG | | | T- | | | | | 0 | | | | | + | | | | | | | |
| CYTB | 9863 | | 10987 | | 1125 | | 374 | | | | ATG | | | TAA | | | | | 7 | | | | | + | | | | | | | |
| tRNA(Ser) | 10995 | | 11062 | | 68 | |  | | | |  | | |  | | | | | 629 | | | | | + | | | | | | | |
| ND1 | 11692 | | 12639 | | 948 | | 315 | | | | GTA | | | TAA | | | | | 0 | | | | | - | | | | | | | |
| tRNA(Leu) | 12640 | | 12708 | | 69 | |  | | | |  | | |  | | | | | 12 | | | | | - | | | | | | | |
| 16S rRNA | 12721 | | 14079 | | 1359 | |  | | | |  | | |  | | | | | 24 | | | | | - | | | | | | | |
| tRNA(Val) | 14104 | | 14174 | | 71 | |  | | | |  | | |  | | | | | 6 | | | | | - | | | | | | | |
| 12S rRNA | 14181 | | 14966 | | 786 | |  | | | |  | | |  | | | | | 658 | | | | | - | | | | | | | |
| tRNA(Met) | 15625 | | 15693 | | 69 | |  | | | |  | | |  | | | | | 6 | | | | | + | | | | | | | |
| tRNA(Ile) | 15700 | | 15765 | | 66 | |  | | | |  | | |  | | | | | 118 | | | | | + | | | | | | | |
| tRNA(Gln) | 15884 | | 15956 | | 73 | |  | | | |  | | |  | | | | | 57 | | | | | - | | | | | | | |
| ND2 | 16014 | | 16979 | | 966 | | 321 | | | | ATA | | | TAA | | | | | 9 | | | | | + | | | | | | | |
| tRNA(Trp) | 16989 | | 17058 | | 70 | |  | | | |  | | |  | | | | | 17 | | | | | + | | | | | | | |
| tRNA(Cys) | 17076 | | 17148 | | 73 | |  | | | |  | | |  | | | | | 72 | | | | | - | | | | | | | |
| tRNA(Tyr) | 17221 | | 17288 | | 68 | |  | | | |  | | |  | | | | | 74 | | | | | - | | | | | | | |
| 5 |  | |  | | ***Pseudomyrmex feralis*** | | | | | | | | |  | | | | |  | | | | |  | | | | | | | |
| **Gene** | **Position** | | | | **Size** | | | | | | **Codon** | | | | | | | | **Intergenic** | | | | |  | | | | | | | |
|  | **From** | | **To** | | **Nucleotide** | | **Aminoacid** | | | | **Start** | | | **Stop** | | | | | **nucleotide** | | | | |  | | | | | | | |
| COX1 | 1 | | 1533 | | 1533 | | 510 | | | | ATG | | | TAA | | | | | 1 | | | | | + | | | | | | | |
| tRNA(Leu) | 1535 | | 1605 | | 71 | |  | | | |  | | |  | | | | | 0 | | | | | + | | | | | | | |
| COX2 | 1606 | | 2283 | | 678 | | 225 | | | | ATT | | | TAA | | | | | 166 | | | | | + | | | | | | | |
| tRNA(Lys) | 2450 | | 2523 | | 74 | |  | | | |  | | |  | | | | | 14 | | | | | + | | | | | | | |
| tRNA(Asp) | 2538 | | 2605 | | 68 | |  | | | |  | | |  | | | | | 0 | | | | | + | | | | | | | |
| ATP8 | 2606 | | 2836 | | 231 | | 76 | | | | ATA | | | TAA | | | | | 426 | | | | | + | | | | | | | |
| ATP6 | 3263 | | 3931 | | 669 | | 222 | | | | ATG | | | TAA | | | | | 27 | | | | | + | | | | | | | |
| COX3 | 3959 | | 4744 | | 786 | | 261 | | | | ATG | | | TAA | | | | | 175 | | | | | + | | | | | | | |
| tRNA(Gly) | 4920 | | 4992 | | 73 | |  | | | |  | | |  | | | | | 3 | | | | | + | | | | | | | |
| ND3 | 4996 | | 5343 | | 348 | | 115 | | | | ATG | | | TAA | | | | | 302 | | | | | + | | | | | | | |
| tRNA(Ala) | 5646 | | 5709 | | 64 | |  | | | |  | | |  | | | | | 0 | | | | | + | | | | | | | |
| tRNA(Arg) | 5710 | | 5776 | | 67 | |  | | | |  | | |  | | | | | 72 | | | | | + | | | | | | | |
| tRNA(Asn) | 5849 | | 5921 | | 73 | |  | | | |  | | |  | | | | | 119 | | | | | + | | | | | | | |
| tRNA(Ser) | 6041 | | 6101 | | 61 | |  | | | |  | | |  | | | | | 225 | | | | | + | | | | | | | |
| tRNA(Glu) | 6327 | | 6400 | | 74 | |  | | | |  | | |  | | | | | 105 | | | | | + | | | | | | | |
| tRNA(Phe) | 6506 | | 6573 | | 68 | |  | | | |  | | |  | | | | | 79 | | | | | - | | | | | | | |
| ND5 | 6653 | | 8314 | | 1662 | | 553 | | | | ATT | | | TAA | | | | | 0 | | | | | - | | | | | | | |
| tRNA(His) | 8315 | | 8383 | | 69 | |  | | | |  | | |  | | | | | 11 | | | | | - | | | | | | | |
| ND4 | 8395 | | 9717 | | 1323 | | 440 | | | | ATG | | | TAG | | | | | 52 | | | | | - | | | | | | | |
| ND4L | 9770 | | 10054 | | 285 | | 94 | | | | ATA | | | TAG | | | | | 294 | | | | | - | | | | | | | |
| tRNA(Thr) | 10349 | | 10423 | | 75 | |  | | | |  | | |  | | | | | 361 | | | | | + | | | | | | | |
| tRNA(Pro) | 10785 | | 10853 | | 69 | |  | | | |  | | |  | | | | | 87 | | | | | - | | | | | | | |
| ND6 | 10941 | | 11474 | | 534 | | 177 | | | | ATG | | | TAA | | | | | 7 | | | | | + | | | | | | | |
| CYTB | 11482 | | 12600 | | 1119 | | 372 | | | | ATG | | | TAA | | | | | 13 | | | | | + | | | | | | | |
| tRNA(Ser) | 12614 | | 12682 | | 69 | |  | | | |  | | |  | | | | | 139 | | | | | + | | | | | | | |
| ND1 | 12822 | | 13766 | | 945 | | 314 | | | | ATT | | | TAA | | | | | 0 | | | | | - | | | | | | | |
| tRNA(Leu) | 13767 | | 13834 | | 68 | |  | | | |  | | |  | | | | | 0 | | | | | - | | | | | | | |
| 16S rRNA | 13835 | | 15174 | | 1340 | |  | | | |  | | |  | | | | | 0 | | | | | - | | | | | | | |
| tRNA(Val) | 15175 | | 15246 | | 72 | |  | | | |  | | |  | | | | | 7 | | | | | - | | | | | | | |
| 12S rRNA | 15254 | | 16049 | | 796 | |  | | | |  | | |  | | | | | 566 | | | | | - | | | | | | | |
| tRNA(Met) | 16616 | | 16686 | | 71 | |  | | | |  | | |  | | | | | 10 | | | | | + | | | | | | | |
| tRNA(Ile) | 16697 | | 16767 | | 71 | |  | | | |  | | |  | | | | | 161 | | | | | + | | | | | | | |
| tRNA(Gln) | 16929 | | 17000 | | 72 | |  | | | |  | | |  | | | | | 69 | | | | | - | | | | | | | |
| ND2 | 17070 | | 18038 | | 969 | | 322 | | | | ATA | | | TAA | | | | | 2 | | | | | + | | | | | | | |
| tRNA(Trp) | 18041 | | 18111 | | 71 | |  | | | |  | | |  | | | | | 229 | | | | | + | | | | | | | |
| tRNA(Cys) | 18341 | | 18412 | | 72 | |  | | | |  | | |  | | | | | 137 | | | | | - | | | | | | | |
| tRNA(Tyr) | 18550 | | 18618 | | 69 | |  | | | |  | | |  | | | | | 217 | | | | | - | | | | | | | |
| 6 |  |  | | ***Pseudomyrmex ferrugineus*** | | | | | | | | |  | | | | |  | | | | |  | | | | | | |  | |
| **Gene** | **Position** | | | **Size** | | | | | **Codon** | | | | | | | | | **Intergenic** | | | | |  | | | | | | |  | |
|  | **From** | **To** | | **Nucleotide** | | **Aminoacid** | | | | **Start** | | | **Stop** | | | | | **nucleotide** | | | | |  | | | | | | |  | |
| COX1 | 1 | 1533 | | 1533 | | 510 | | | | ATG | | | TAA | | | | | 7 | | | | | + | | | | | | |  | |
| tRNA(Leu) | 1541 | 1611 | | 71 | |  | | | |  | | |  | | | | | 0 | | | | | + | | | | | | |  | |
| COX2 | 1612 | 2289 | | 678 | | 225 | | | | ATT | | | TAA | | | | | 399 | | | | | + | | | | | | |  | |
| tRNA(Lys) | 2689 | 2760 | | 72 | |  | | | |  | | |  | | | | | 6 | | | | | + | | | | | | |  | |
| tRNA(Asp) | 2767 | 2833 | | 67 | |  | | | |  | | |  | | | | | 0 | | | | | + | | | | | | |  | |
| ATP8 | 2834 | 3049 | | 216 | | 71 | | | | ATA | | | TAA | | | | | 481 | | | | | + | | | | | | |  | |
| ATP6 | 3531 | 4199 | | 669 | | 222 | | | | ATG | | | TAA | | | | | 46 | | | | | + | | | | | | |  | |
| COX3 | 4246 | 5028 | | 783 | | 260 | | | | ATG | | | TAA | | | | | 274 | | | | | + | | | | | | |  | |
| tRNA(Gly) | 5303 | 5371 | | 69 | |  | | | |  | | |  | | | | | 3 | | | | | + | | | | | | |  | |
| ND3 | 5375 | 5722 | | 348 | | 115 | | | | ATG | | | TAA | | | | | 92 | | | | | + | | | | | | |  | |
| tRNA(Ala) | 5815 | 5889 | | 75 | |  | | | |  | | |  | | | | | 0 | | | | | + | | | | | | |  | |
| tRNA(Arg) | 5890 | 5954 | | 65 | |  | | | |  | | |  | | | | | 53 | | | | | + | | | | | | |  | |
| tRNA(Asn) | 6008 | 6075 | | 68 | |  | | | |  | | |  | | | | | 316 | | | | | + | | | | | | |  | |
| tRNA(Ser) | 6392 | 6453 | | 62 | |  | | | |  | | |  | | | | | 202 | | | | | + | | | | | | |  | |
| tRNA(Glu) | 6656 | 6727 | | 72 | |  | | | |  | | |  | | | | | 166 | | | | | + | | | | | | |  | |
| tRNA(Phe) | 6894 | 6963 | | 70 | |  | | | |  | | |  | | | | | 0 | | | | | - | | | | | | |  | |
| ND5 | 6964 | 8627 | | 1664 | | 554 | | | | ATA | | | TA- | | | | | 0 | | | | | - | | | | | | |  | |
| tRNA(His) | 8628 | 8696 | | 69 | |  | | | |  | | |  | | | | | 9 | | | | | - | | | | | | |  | |
| ND4 | 8706 | 10031 | | 1326 | | 441 | | | | ATG | | | TAA | | | | | 47 | | | | | - | | | | | | |  | |
| ND4L | 10079 | 10363 | | 285 | | 94 | | | | ATA | | | TAA | | | | | 99 | | | | | - | | | | | | |  | |
| tRNA(Thr) | 10463 | 10534 | | 72 | |  | | | |  | | |  | | | | | 83 | | | | | + | | | | | | |  | |
| tRNA(Pro) | 10618 | 10686 | | 69 | |  | | | |  | | |  | | | | | 63 | | | | | - | | | | | | |  | |
| ND6 | 10750 | 11283 | | 534 | | 177 | | | | ATG | | | TAA | | | | | 12 | | | | | + | | | | | | |  | |
| CYTB | 11296 | 12411 | | 1116 | | 371 | | | | ATG | | | TAA | | | | | 18 | | | | | + | | | | | | |  | |
| tRNA(Ser) | 12430 | 12498 | | 69 | |  | | | |  | | |  | | | | | 101 | | | | | + | | | | | | |  | |
| ND1 | 12600 | 13544 | | 945 | | 314 | | | | GTT | | | TAA | | | | | 0 | | | | | - | | | | | | |  | |
| tRNA(Leu) | 13545 | 13616 | | 72 | |  | | | |  | | |  | | | | | 0 | | | | | - | | | | | | |  | |
| 16S rRNA | 13617 | 14956 | | 1340 | |  | | | |  | | |  | | | | | 0 | | | | | - | | | | | | |  | |
| tRNA(Val) | 14957 | 15027 | | 71 | |  | | | |  | | |  | | | | | 46 | | | | | - | | | | | | |  | |
| 12S rRNA | 15074 | 15866 | | 793 | |  | | | |  | | |  | | | | | 572 | | | | | - | | | | | | |  | |
| tRNA(Met) | 16439 | 16511 | | 73 | |  | | | |  | | |  | | | | | 8 | | | | | + | | | | | | |  | |
| tRNA(Ile) | 16520 | 16587 | | 68 | |  | | | |  | | |  | | | | | 94 | | | | | + | | | | | | |  | |
| tRNA(Gln) | 16682 | 16750 | | 69 | |  | | | |  | | |  | | | | | 65 | | | | | - | | | | | | |  | |
| ND2 | 16816 | 17784 | | 969 | | 322 | | | | ATA | | | TAA | | | | | 0 | | | | | + | | | | | | |  | |
| tRNA(Trp) | 17785 | 17853 | | 69 | |  | | | |  | | |  | | | | | 140 | | | | | + | | | | | | |  | |
| tRNA(Cys) | 17994 | 18060 | | 67 | |  | | | |  | | |  | | | | | 106 | | | | | - | | | | | | |  | |
| tRNA(Tyr) | 18167 | 18236 | | 70 | |  | | | |  | | |  | | | | | 244 | | | | | - | | | | | | |  | |
| 7 |  |  | | ***Pseudomyrmex flavicornis*** | | | | | | | | |  | | | | |  | | | | |  | | | | | | |  | |
| **Gene** | **Position** | | | **Size** | | | | | **Codon** | | | | | | | | | **Intergenic** | | | | |  | | | | | | |  | |
|  | **From** | **To** | | **Nucleotide** | | **Aminoacid** | | | | **Start** | | | **Stop** | | | | | **nucleotide** | | | | |  | | | | | | |  | |
| COX1 | 1 | 1533 | | 1533 | | 510 | | | | ATG | | | TAA | | | | | 7 | | | | | + | | | | | | |  | |
| tRNA(Leu) | 1541 | 1611 | | 71 | |  | | | |  | | |  | | | | | 0 | | | | | + | | | | | | |  | |
| COX2 | 1612 | 2289 | | 678 | | 225 | | | | ATT | | | TAA | | | | | 412 | | | | | + | | | | | | |  | |
| tRNA(Lys) | 2702 | 2773 | | 72 | |  | | | |  | | |  | | | | | 6 | | | | | + | | | | | | |  | |
| tRNA(Asp) | 2780 | 2848 | | 69 | |  | | | |  | | |  | | | | | 0 | | | | | + | | | | | | |  | |
| ATP8 | 2849 | 3064 | | 216 | | 71 | | | | ATT | | | TAA | | | | | 474 | | | | | + | | | | | | |  | |
| ATP6 | 3539 | 4207 | | 669 | | 222 | | | | ATG | | | TAA | | | | | 46 | | | | | + | | | | | | |  | |
| COX3 | 4254 | 5036 | | 783 | | 260 | | | | ATG | | | TAG | | | | | 272 | | | | | + | | | | | | |  | |
| tRNA(Gly) | 5309 | 5377 | | 69 | |  | | | |  | | |  | | | | | 3 | | | | | + | | | | | | |  | |
| ND3 | 5381 | 5728 | | 348 | | 115 | | | | ATG | | | TAA | | | | | 94 | | | | | + | | | | | | |  | |
| tRNA(Ala) | 5823 | 5901 | | 79 | |  | | | |  | | |  | | | | | -2 | | | | | + | | | | | | |  | |
| tRNA(Arg) | 5900 | 5968 | | 69 | |  | | | |  | | |  | | | | | 51 | | | | | + | | | | | | |  | |
| tRNA(Asn) | 6020 | 6087 | | 68 | |  | | | |  | | |  | | | | | 306 | | | | | + | | | | | | |  | |
| tRNA(Ser) | 6394 | 6455 | | 62 | |  | | | |  | | |  | | | | | 209 | | | | | + | | | | | | |  | |
| tRNA(Glu) | 6665 | 6736 | | 72 | |  | | | |  | | |  | | | | | 165 | | | | | + | | | | | | |  | |
| tRNA(Phe) | 6902 | 6968 | | 67 | |  | | | |  | | |  | | | | | 0 | | | | | - | | | | | | |  | |
| ND5 | 6969 | 8632 | | 1664 | | 554 | | | | ATA | | | TA- | | | | | 0 | | | | | - | | | | | | |  | |
| tRNA(His) | 8633 | 8701 | | 69 | |  | | | |  | | |  | | | | | 9 | | | | | - | | | | | | |  | |
| ND4 | 8711 | 10036 | | 1326 | | 441 | | | | ATG | | | TAA | | | | | 47 | | | | | - | | | | | | |  | |
| ND4L | 10084 | 10368 | | 285 | | 94 | | | | ATA | | | AAT | | | | | 98 | | | | | - | | | | | | |  | |
| tRNA(Thr) | 10467 | 10538 | | 72 | |  | | | |  | | |  | | | | | 80 | | | | | + | | | | | | |  | |
| tRNA(Pro) | 10619 | 10687 | | 69 | |  | | | |  | | |  | | | | | 70 | | | | | - | | | | | | |  | |
| ND6 | 10758 | 11291 | | 534 | | 177 | | | | ATG | | | TAA | | | | | 12 | | | | | + | | | | | | |  | |
| CYTB | 11304 | 12419 | | 1116 | | 371 | | | | ATG | | | TAA | | | | | 20 | | | | | + | | | | | | |  | |
| tRNA(Ser) | 12440 | 12510 | | 71 | |  | | | |  | | |  | | | | | 102 | | | | | + | | | | | | |  | |
| ND1 | 12613 | 13557 | | 945 | | 314 | | | | GTT | | | TAA | | | | | 0 | | | | | - | | | | | | |  | |
| tRNA(Leu) | 13558 | 13629 | | 72 | |  | | | |  | | |  | | | | | 0 | | | | | - | | | | | | |  | |
| 16S rRNA | 13630 | 14968 | | 1339 | |  | | | |  | | |  | | | | | 0 | | | | | - | | | | | | |  | |
| tRNA(Val) | 14969 | 15039 | | 71 | |  | | | |  | | |  | | | | | 44 | | | | | - | | | | | | |  | |
| 12S rRNA | 15084 | 15879 | | 796 | |  | | | |  | | |  | | | | | 570 | | | | | - | | | | | | |  | |
| tRNA(Met) | 16450 | 16522 | | 73 | |  | | | |  | | |  | | | | | 8 | | | | | + | | | | | | |  | |
| tRNA(Ile) | 16531 | 16598 | | 68 | |  | | | |  | | |  | | | | | 88 | | | | | + | | | | | | |  | |
| tRNA(Gln) | 16687 | 16755 | | 69 | |  | | | |  | | |  | | | | | 67 | | | | | - | | | | | | |  | |
| ND2 | 16823 | 17791 | | 969 | | 322 | | | | ATA | | | TAA | | | | | 0 | | | | | + | | | | | | |  | |
| tRNA(Trp) | 17792 | 17860 | | 69 | |  | | | |  | | |  | | | | | 142 | | | | | + | | | | | | |  | |
| tRNA(Cys) | 18003 | 18069 | | 67 | |  | | | |  | | |  | | | | | 111 | | | | | - | | | | | | |  | |
| tRNA(Tyr) | 18181 | 18250 | | 70 | |  | | | |  | | |  | | | | | 248 | | | | | - | | | | | | |  | |
| 8 |  |  | | ***Pseudomyrmex janzeni*** | | | | | | | | |  | | | | |  | | | | |  | | | | | | |  | |
| **Gene** | **Position** | | | **Size** | | | | | **Codon** | | | | | | | | | **Intergenic** | | | | |  | | | | | | |  | |
|  | **From** | **To** | | **Nucleotide** | | **Aminoacid** | | | | **Start** | | | **Stop** | | | | | **nucleotide** | | | | |  | | | | | | |  | |
| COX1 | 1 | 1533 | | 1533 | | 510 | | | | ATG | | | TAA | | | | | 7 | | | | | + | | | | | | |  | |
| tRNA(Leu) | 1541 | 1611 | | 71 | |  | | | |  | | |  | | | | | 0 | | | | | + | | | | | | |  | |
| COX2 | 1612 | 2289 | | 678 | | 225 | | | | ATT | | | TAA | | | | | 337 | | | | | + | | | | | | |  | |
| tRNA(Lys) | 2627 | 2698 | | 72 | |  | | | |  | | |  | | | | | 6 | | | | | + | | | | | | |  | |
| tRNA(Asp) | 2705 | 2773 | | 69 | |  | | | |  | | |  | | | | | 0 | | | | | + | | | | | | |  | |
| ATP8 | 2774 | 2989 | | 216 | | 71 | | | | ATC | | | TAA | | | | | 481 | | | | | + | | | | | | |  | |
| ATP6 | 3471 | 4139 | | 669 | | 222 | | | | ATG | | | TAA | | | | | 38 | | | | | + | | | | | | |  | |
| COX3 | 4178 | 4960 | | 783 | | 260 | | | | ATG | | | TAA | | | | | 217 | | | | | + | | | | | | |  | |
| tRNA(Gly) | 5178 | 5246 | | 69 | |  | | | |  | | |  | | | | | 3 | | | | | + | | | | | | |  | |
| ND3 | 5250 | 5597 | | 348 | | 115 | | | | ATG | | | TAA | | | | | 95 | | | | | + | | | | | | |  | |
| tRNA(Ala) | 5693 | 5771 | | 79 | |  | | | |  | | |  | | | | | -2 | | | | | + | | | | | | |  | |
| tRNA(Arg) | 5770 | 5836 | | 67 | |  | | | |  | | |  | | | | | 46 | | | | | + | | | | | | |  | |
| tRNA(Asn) | 5883 | 5960 | | 78 | |  | | | |  | | |  | | | | | 307 | | | | | + | | | | | | |  | |
| tRNA(Ser) | 6268 | 6329 | | 62 | |  | | | |  | | |  | | | | | 194 | | | | | + | | | | | | |  | |
| tRNA(Glu) | 6524 | 6595 | | 72 | |  | | | |  | | |  | | | | | 172 | | | | | + | | | | | | |  | |
| tRNA(Phe) | 6768 | 6834 | | 67 | |  | | | |  | | |  | | | | | 0 | | | | | - | | | | | | |  | |
| ND5 | 6835 | 8498 | | 1664 | | 554 | | | | ATA | | | TA- | | | | | 0 | | | | | - | | | | | | |  | |
| tRNA(His) | 8499 | 8565 | | 67 | |  | | | |  | | |  | | | | | 10 | | | | | - | | | | | | |  | |
| ND4 | 8576 | 9901 | | 1326 | | 441 | | | | ATG | | | TAA | | | | | 48 | | | | | - | | | | | | |  | |
| ND4L | 9950 | 10234 | | 285 | | 94 | | | | ATA | | | TAA | | | | | 91 | | | | | - | | | | | | |  | |
| tRNA(Thr) | 10326 | 10397 | | 72 | |  | | | |  | | |  | | | | | 75 | | | | | + | | | | | | |  | |
| tRNA(Pro) | 10473 | 10541 | | 69 | |  | | | |  | | |  | | | | | 65 | | | | | - | | | | | | |  | |
| ND6 | 10607 | 11140 | | 534 | | 177 | | | | ATG | | | TAA | | | | | 14 | | | | | + | | | | | | |  | |
| CYTB | 11155 | 12270 | | 1116 | | 371 | | | | ATG | | | TAA | | | | | 20 | | | | | + | | | | | | |  | |
| tRNA(Ser) | 12291 | 12359 | | 69 | |  | | | |  | | |  | | | | | 102 | | | | | + | | | | | | |  | |
| ND1 | 12462 | 13406 | | 945 | | 314 | | | |  | | | TAA | | | | | 0 | | | | | - | | | | | | |  | |
| tRNA(Leu) | 13407 | 13478 | | 72 | |  | | | |  | | |  | | | | | 0 | | | | | - | | | | | | |  | |
| 16S rRNA | 13479 | 14816 | | 1338 | |  | | | |  | | |  | | | | | 0 | | | | | - | | | | | | |  | |
| tRNA(Val) | 14817 | 14887 | | 71 | |  | | | |  | | |  | | | | | 44 | | | | | - | | | | | | |  | |
| 12S rRNA | 14932 | 15726 | | 795 | |  | | | |  | | |  | | | | | 571 | | | | | - | | | | | | |  | |
| tRNA(Met) | 16298 | 16370 | | 73 | |  | | | |  | | |  | | | | | 8 | | | | | + | | | | | | |  | |
| tRNA(Ile) | 16379 | 16446 | | 68 | |  | | | |  | | |  | | | | | 93 | | | | | + | | | | | | |  | |
| tRNA(Gln) | 16540 | 16608 | | 69 | |  | | | |  | | |  | | | | | 67 | | | | | - | | | | | | |  | |
| ND2 | 16676 | 17644 | | 969 | | 322 | | | | ATA | | | TAA | | | | | 0 | | | | | + | | | | | | |  | |
| tRNA(Trp) | 17645 | 17713 | | 69 | |  | | | |  | | |  | | | | | 143 | | | | | + | | | | | | |  | |
| tRNA(Cys) | 17857 | 17923 | | 67 | |  | | | |  | | |  | | | | | 121 | | | | | - | | | | | | |  | |
| tRNA(Tyr) | 18045 | 18114 | | 70 | |  | | | |  | | |  | | | | | 266 | | | | | - | | | | | | |  | |
| 9 |  |  | | ***Pseudomyrmex palidus*** | | | | | | | | |  | | | | |  | | | | |  | | | | | | |  | |
| **Gene** | **Position** | | | **Size** | | | | | **Codon** | | | | | | | | | **Intergenic** | | | | |  | | | | | | |  | |
|  | **From** | **To** | | **Nucleotide** | | **Aminoacid** | | | | **Start** | | | **Stop** | | | | | **nucleotide** | | | | |  | | | | | | |  | |
| COX1 | 1 | 1533 | | 1533 | | 510 | | | | ATG | | | TAA | | | | | 47 | | | | | + | | | | | | |  | |
| tRNA(Leu) | 1581 | 1649 | | 69 | |  | | | |  | | |  | | | | | 0 | | | | | + | | | | | | |  | |
| COX2 | 1650 | 2330 | | 681 | | 226 | | | | ATT | | | TAA | | | | | 84 | | | | | + | | | | | | |  | |
| tRNA(Lys) | 2415 | 2485 | | 71 | |  | | | |  | | |  | | | | | 31 | | | | | + | | | | | | |  | |
| tRNA(Asp) | 2517 | 2590 | | 74 | |  | | | |  | | |  | | | | | 0 | | | | | + | | | | | | |  | |
| ATP8 | 2591 | 2767 | | 177 | | 58 | | | | ATA | | | TAA | | | | | 116 | | | | | + | | | | | | |  | |
| ATP6 | 2884 | 3552 | | 669 | | 222 | | | | ATG | | | TAA | | | | | 87 | | | | | + | | | | | | |  | |
| COX3 | 3640 | 4419 | | 780 | | 259 | | | | ATG | | | TAG | | | | | 117 | | | | | + | | | | | | |  | |
| tRNA(Gly) | 4537 | 4609 | | 73 | |  | | | |  | | |  | | | | | 5 | | | | | + | | | | | | |  | |
| ND3 | 4615 | 4962 | | 348 | | 115 | | | | ATG | | | TAA | | | | | 70 | | | | | + | | | | | | |  | |
| tRNA(Ala) | 5033 | 5099 | | 67 | |  | | | |  | | |  | | | | | 0 | | | | | + | | | | | | |  | |
| tRNA(Arg) | 5100 | 5164 | | 65 | |  | | | |  | | |  | | | | | 25 | | | | | + | | | | | | |  | |
| tRNA(Asn) | 5190 | 5256 | | 67 | |  | | | |  | | |  | | | | | 191 | | | | | + | | | | | | |  | |
| tRNA(Ser) | 5448 | 5508 | | 61 | |  | | | |  | | |  | | | | | 108 | | | | | + | | | | | | |  | |
| tRNA(Glu) | 5617 | 5689 | | 73 | |  | | | |  | | |  | | | | | 107 | | | | | + | | | | | | |  | |
| tRNA(Phe) | 5797 | 5865 | | 69 | |  | | | |  | | |  | | | | | 6 | | | | | - | | | | | | |  | |
| ND5 | 5872 | 7545 | | 1674 | | 557 | | | | ATT | | | TAG | | | | | 0 | | | | | - | | | | | | |  | |
| tRNA(His) | 7546 | 7610 | | 65 | |  | | | |  | | |  | | | | | 8 | | | | | - | | | | | | |  | |
| ND4 | 7619 | 8953 | | 1335 | | 444 | | | | ATG | | | TAA | | | | | 0 | | | | | - | | | | | | |  | |
| ND4L | 8954 | 9236 | | 283 | | 94 | | | | ATT | | | T- | | | | | 155 | | | | | - | | | | | | |  | |
| tRNA(Thr) | 9392 | 9461 | | 70 | |  | | | |  | | |  | | | | | 21 | | | | | + | | | | | | |  | |
| tRNA(Pro) | 9483 | 9551 | | 69 | |  | | | |  | | |  | | | | | 41 | | | | | - | | | | | | |  | |
| ND6 | 9593 | 10121 | | 529 | | 176 | | | | ATG | | | T- | | | | | 0 | | | | | + | | | | | | |  | |
| CYTB | 10122 | 11240 | | 1119 | | 372 | | | | ATG | | | TAA | | | | | 11 | | | | | + | | | | | | |  | |
| tRNA(Ser) | 11252 | 11320 | | 69 | |  | | | |  | | |  | | | | | 184 | | | | | + | | | | | | |  | |
| ND1 | 11505 | 12449 | | 945 | | 314 | | | | ATA | | | TAA | | | | | 0 | | | | | - | | | | | | |  | |
| tRNA(Leu) | 12450 | 12517 | | 68 | |  | | | |  | | |  | | | | | 0 | | | | | - | | | | | | |  | |
| 16S rRNA | 12518 | 13853 | | 1336 | |  | | | |  | | |  | | | | | 0 | | | | | - | | | | | | |  | |
| tRNA(Val) | 13854 | 13924 | | 71 | |  | | | |  | | |  | | | | | 7 | | | | | - | | | | | | |  | |
| 12S rRNA | 13932 | 14726 | | 795 | |  | | | |  | | |  | | | | | 650 | | | | | - | | | | | | |  | |
| tRNA(Met) | 15377 | 15444 | | 68 | |  | | | |  | | |  | | | | | 2 | | | | | + | | | | | | |  | |
| tRNA(Ile) | 15447 | 15512 | | 66 | |  | | | |  | | |  | | | | | 9 | | | | | + | | | | | | |  | |
| tRNA(Gln) | 15522 | 15593 | | 72 | |  | | | |  | | |  | | | | | 53 | | | | | - | | | | | | |  | |
| ND2 | 15647 | 16615 | | 969 | | 322 | | | | ATA | | | TAA | | | | | 17 | | | | | + | | | | | | |  | |
| tRNA(Trp) | 16633 | 16703 | | 71 | |  | | | |  | | |  | | | | | 25 | | | | | + | | | | | | |  | |
| tRNA(Cys) | 16729 | 16795 | | 67 | |  | | | |  | | |  | | | | | 99 | | | | | - | | | | | | |  | |
| tRNA(Tyr) | 16895 | 16959 | | 65 | |  | | | |  | | |  | | | | | 158 | | | | | - | | | | | | |  | |
| 10 |  |  | | ***Pseudomyrmex particeps*** | | | | | | | | |  | | | | |  | | | | |  | | | | | | |  | |
| **Gene** | **Position** | | | **Size** | | | | | **Codon** | | | | | | | | | **Intergenic** | | | | |  | | | | | | |  | |
|  | **From** | **To** | | **Nucleotide** | | **Aminoacid** | | | | **Start** | | | **Stop** | | | | | **nucleotide** | | | | |  | | | | | | |  | |
| COX1 | 1 | 1533 | | 1533 | | 510 | | | | ATG | | | TAA | | | | | 5 | | | | | + | | | | | | |  | |
| tRNA(Leu) | 1539 | 1610 | | 72 | |  | | | |  | | |  | | | | | 0 | | | | | + | | | | | | |  | |
| COX2 | 1611 | 2291 | | 681 | | 226 | | | | ATT | | | TAA | | | | | 219 | | | | | + | | | | | | |  | |
| tRNA(Lys) | 2511 | 2580 | | 70 | |  | | | |  | | |  | | | | | 4 | | | | | + | | | | | | |  | |
| tRNA(Asp) | 2585 | 2651 | | 67 | |  | | | |  | | |  | | | | | 0 | | | | | + | | | | | | |  | |
| ATP8 | 2652 | 2849 | | 198 | | 65 | | | | ATA | | | TAG | | | | | 363 | | | | | + | | | | | | |  | |
| ATP6 | 3213 | 3881 | | 669 | | 222 | | | | ATG | | | TAA | | | | | 10 | | | | | + | | | | | | |  | |
| COX3 | 3892 | 4671 | | 780 | | 259 | | | | ATG | | | TAA | | | | | 177 | | | | | + | | | | | | |  | |
| tRNA(Gly) | 4849 | 4915 | | 67 | |  | | | |  | | |  | | | | | 0 | | | | | + | | | | | | |  | |
| ND3 | 4916 | 5263 | | 348 | | 115 | | | | ATA | | | TAA | | | | | 468 | | | | | + | | | | | | |  | |
| tRNA(Ala) | 5732 | 5801 | | 70 | |  | | | |  | | |  | | | | | -2 | | | | | + | | | | | | |  | |
| tRNA(Arg) | 5800 | 5866 | | 67 | |  | | | |  | | |  | | | | | 33 | | | | | + | | | | | | |  | |
| tRNA(Asn) | 5900 | 5970 | | 71 | |  | | | |  | | |  | | | | | 169 | | | | | + | | | | | | |  | |
| tRNA(Ser) | 6140 | 6199 | | 60 | |  | | | |  | | |  | | | | | 151 | | | | | + | | | | | | |  | |
| tRNA(Glu) | 6351 | 6425 | | 75 | |  | | | |  | | |  | | | | | 36 | | | | | + | | | | | | |  | |
| tRNA(Phe) | 6462 | 6527 | | 66 | |  | | | |  | | |  | | | | | 4 | | | | | - | | | | | | |  | |
| ND5 | 6532 | 8196 | | 1665 | | 554 | | | | ATA | | | TAA | | | | | 0 | | | | | - | | | | | | |  | |
| tRNA(His) | 8197 | 8268 | | 72 | |  | | | |  | | |  | | | | | 11 | | | | | - | | | | | | |  | |
| ND4 | 8280 | 9602 | | 1323 | | 440 | | | | ATG | | | TAG | | | | | 23 | | | | | - | | | | | | |  | |
| ND4L | 9626 | 9910 | | 285 | | 94 | | | | ATA | | | TAA | | | | | 189 | | | | | - | | | | | | |  | |
| tRNA(Thr) | 10100 | 10166 | | 67 | |  | | | |  | | |  | | | | | 353 | | | | | + | | | | | | |  | |
| tRNA(Pro) | 10520 | 10591 | | 72 | |  | | | |  | | |  | | | | | 77 | | | | | - | | | | | | |  | |
| ND6 | 10669 | 11205 | | 537 | | 178 | | | | ATG | | | TAA | | | | | 7 | | | | | + | | | | | | |  | |
| CYTB | 11213 | 12331 | | 1119 | | 372 | | | | ATG | | | TAA | | | | | 30 | | | | | + | | | | | | |  | |
| tRNA(Ser) | 12362 | 12430 | | 69 | |  | | | |  | | |  | | | | | 48 | | | | | + | | | | | | |  | |
| ND1 | 12479 | 13423 | | 945 | | 314 | | | | ATT | | | TAA | | | | | 0 | | | | | - | | | | | | |  | |
| tRNA(Leu) | 13424 | 13498 | | 75 | |  | | | |  | | |  | | | | | 0 | | | | | - | | | | | | |  | |
| 16S rRNA | 13499 | 14823 | | 1325 | |  | | | |  | | |  | | | | | 0 | | | | | - | | | | | | |  | |
| tRNA(Val) | 14824 | 14892 | | 69 | |  | | | |  | | |  | | | | | 27 | | | | | - | | | | | | |  | |
| 12S rRNA | 14920 | 15692 | | 773 | |  | | | |  | | |  | | | | | 562 | | | | | - | | | | | | |  | |
| tRNA(Met) | 16255 | 16323 | | 69 | |  | | | |  | | |  | | | | | 10 | | | | | + | | | | | | |  | |
| tRNA(Ile) | 16334 | 16398 | | 65 | |  | | | |  | | |  | | | | | 264 | | | | | + | | | | | | |  | |
| tRNA(Gln) | 16663 | 16735 | | 73 | |  | | | |  | | |  | | | | | 67 | | | | | - | | | | | | |  | |
| ND2 | 16803 | 17769 | | 967 | | 322 | | | | ATA | | | T- | | | | | 0 | | | | | + | | | | | | |  | |
| tRNA(Trp) | 17770 | 17837 | | 68 | |  | | | |  | | |  | | | | | 192 | | | | | + | | | | | | |  | |
| tRNA(Cys) | 18030 | 18098 | | 69 | |  | | | |  | | |  | | | | | 106 | | | | | - | | | | | | |  | |
| tRNA(Tyr) | 18205 | 18275 | | 71 | |  | | | |  | | |  | | | | | 249 | | | | | - | | | | | | |  | |
| 11 |  |  | | ***Pseudomyrmex peperi*** | | | | | | | | |  | | | | |  | | | | |  | | | | | | |  | |
| **Gene** | **Position** | | | **Size** | | | | | **Codon** | | | | | | | | | **Intergenic** | | | | |  | | | | | | |  | |
|  | **From** | **To** | | **Nucleotide** | | **Aminoacid** | | | | **Start** | | | **Stop** | | | | | **nucleotide** | | | | |  | | | | | | |  | |
| COX1 | 1 | 1533 | | 1533 | | 510 | | | | ATG | | | TAA | | | | | 5 | | | | | + | | | | | | |  | |
| tRNA(Leu) | 1539 | 1608 | | 70 | |  | | | |  | | |  | | | | | 0 | | | | | + | | | | | | |  | |
| COX2 | 1609 | 2280 | | 672 | | 223 | | | | ATT | | | TAA | | | | | 415 | | | | | + | | | | | | |  | |
| tRNA(Lys) | 2696 | 2764 | | 69 | |  | | | |  | | |  | | | | | 9 | | | | | + | | | | | | |  | |
| tRNA(Asp) | 2774 | 2845 | | 72 | |  | | | |  | | |  | | | | | 3 | | | | | + | | | | | | |  | |
| ATP8 | 2849 | 3058 | | 210 | | 69 | | | | ATA | | | TAG | | | | | 282 | | | | | + | | | | | | |  | |
| ATP6 | 3341 | 4009 | | 669 | | 222 | | | | ATG | | | TAA | | | | | 71 | | | | | + | | | | | | |  | |
| COX3 | 4081 | 4863 | | 783 | | 260 | | | | ATG | | | TAA | | | | | 254 | | | | | + | | | | | | |  | |
| tRNA(Gly) | 5118 | 5186 | | 69 | |  | | | |  | | |  | | | | | 3 | | | | | + | | | | | | |  | |
| ND3 | 5190 | 5537 | | 348 | | 115 | | | | ATG | | | TAA | | | | | 116 | | | | | + | | | | | | |  | |
| tRNA(Ala) | 5654 | 5720 | | 67 | |  | | | |  | | |  | | | | | 0 | | | | | + | | | | | | |  | |
| tRNA(Arg) | 5721 | 5787 | | 67 | |  | | | |  | | |  | | | | | 62 | | | | | + | | | | | | |  | |
| tRNA(Asn) | 5850 | 5920 | | 71 | |  | | | |  | | |  | | | | | 308 | | | | | + | | | | | | |  | |
| tRNA(Ser) | 6229 | 6290 | | 62 | |  | | | |  | | |  | | | | | 232 | | | | | + | | | | | | |  | |
| tRNA(Glu) | 6523 | 6594 | | 72 | |  | | | |  | | |  | | | | | 95 | | | | | + | | | | | | |  | |
| tRNA(Phe) | 6690 | 6757 | | 68 | |  | | | |  | | |  | | | | | 2 | | | | | - | | | | | | |  | |
| ND5 | 6760 | 8421 | | 1662 | | 553 | | | | ATA | | | TAA | | | | | 0 | | | | | - | | | | | | |  | |
| tRNA(His) | 8422 | 8491 | | 70 | |  | | | |  | | |  | | | | | 10 | | | | | - | | | | | | |  | |
| ND4 | 8502 | 9824 | | 1323 | | 440 | | | | ATG | | | TAA | | | | | 45 | | | | | - | | | | | | |  | |
| ND4L | 9870 | 10154 | | 285 | | 94 | | | | ATA | | | TAA | | | | | 203 | | | | | - | | | | | | |  | |
| tRNA(Thr) | 10358 | 10424 | | 67 | |  | | | |  | | |  | | | | | 82 | | | | | + | | | | | | |  | |
| tRNA(Pro) | 10507 | 10574 | | 68 | |  | | | |  | | |  | | | | | 98 | | | | | - | | | | | | |  | |
| ND6 | 10673 | 11206 | | 534 | | 177 | | | | ATG | | | TAA | | | | | 17 | | | | | + | | | | | | |  | |
| CYTB | 11224 | 12348 | | 1125 | | 374 | | | | ATG | | | TAA | | | | | 21 | | | | | + | | | | | | |  | |
| tRNA(Ser) | 12370 | 12439 | | 70 | |  | | | |  | | |  | | | | | 203 | | | | | + | | | | | | |  | |
| ND1 | 12643 | 13590 | | 948 | | 315 | | | | ATA | | | TAG | | | | | 0 | | | | | - | | | | | | |  | |
| tRNA(Leu) | 13591 | 13661 | | 71 | |  | | | |  | | |  | | | | | 0 | | | | | - | | | | | | |  | |
| 16S rRNA | 13662 | 15015 | | 1354 | |  | | | |  | | |  | | | | | 0 | | | | | - | | | | | | |  | |
| tRNA(Val) | 15016 | 15087 | | 72 | |  | | | |  | | |  | | | | | 10 | | | | | - | | | | | | |  | |
| 12S rRNA | 15098 | 15886 | | 789 | |  | | | |  | | |  | | | | | 562 | | | | | - | | | | | | |  | |
| tRNA(Met) | 16449 | 16517 | | 69 | |  | | | |  | | |  | | | | | 4 | | | | | + | | | | | | |  | |
| tRNA(Ile) | 16522 | 16589 | | 68 | |  | | | |  | | |  | | | | | 109 | | | | | + | | | | | | |  | |
| tRNA(Gln) | 16699 | 16772 | | 74 | |  | | | |  | | |  | | | | | 63 | | | | | - | | | | | | |  | |
| ND2 | 16836 | 17806 | | 971 | | 323 | | | | ATA | | | TA- | | | | | 0 | | | | | + | | | | | | |  | |
| tRNA(Trp) | 17807 | 17875 | | 69 | |  | | | |  | | |  | | | | | 199 | | | | | + | | | | | | |  | |
| tRNA(Cys) | 18075 | 18146 | | 72 | |  | | | |  | | |  | | | | | 160 | | | | | - | | | | | | |  | |
| tRNA(Tyr) | 18307 | 18372 | | 66 | |  | | | |  | | |  | | | | |  | | | | | - | | | | | | |  | |
| 12 |  |  | | ***Pseudomyrmex veneficus*** | | | | | | | | |  | | | | |  | | | | |  | | | | | | |  | |
| **Gene** | **Position** | | | **Size** | | | | | **Codon** | | | | | | | | | **Intergenic** | | | | |  | | | | | | |  | |
|  | **From** | **To** | | **Nucleotide** | | **Aminoacid** | | | | **Start** | | | **Stop** | | | | | **nucleotide** | | | | |  | | | | | | |  | |
| COX1 | 1 | 1533 | | 1533 | | 510 | | | | ATG | | | TAA | | | | | 8 | | | | | + | | | | | | |  | |
| tRNA(Leu) | 1542 | 1611 | | 70 | |  | | | |  | | |  | | | | | 0 | | | | | + | | | | | | |  | |
| COX2 | 1612 | 2289 | | 678 | | 225 | | | | ATT | | | TAA | | | | | 199 | | | | | + | | | | | | |  | |
| tRNA(Lys) | 2489 | 2560 | | 72 | |  | | | |  | | |  | | | | | 8 | | | | | + | | | | | | |  | |
| tRNA(Asp) | 2569 | 2641 | | 73 | |  | | | |  | | |  | | | | | 0 | | | | | + | | | | | | |  | |
| ATP8 | 2642 | 2857 | | 216 | | 71 | | | | ATT | | | TAA | | | | | 339 | | | | | + | | | | | | |  | |
| ATP6 | 3197 | 3865 | | 669 | | 222 | | | | ATG | | | TAA | | | | | 57 | | | | | + | | | | | | |  | |
| COX3 | 3923 | 4702 | | 780 | | 259 | | | | ATG | | | TAA | | | | | 187 | | | | | + | | | | | | |  | |
| tRNA(Gly) | 4890 | 4958 | | 69 | |  | | | |  | | |  | | | | | 3 | | | | | + | | | | | | |  | |
| ND3 | 4962 | 5309 | | 348 | | 115 | | | | ATG | | | TAA | | | | | 106 | | | | | + | | | | | | |  | |
| tRNA(Ala) | 5416 | 5490 | | 75 | |  | | | |  | | |  | | | | | 0 | | | | | + | | | | | | |  | |
| tRNA(Arg) | 5491 | 5556 | | 66 | |  | | | |  | | |  | | | | | 52 | | | | | + | | | | | | |  | |
| tRNA(Asn) | 5609 | 5683 | | 75 | |  | | | |  | | |  | | | | | 328 | | | | | + | | | | | | |  | |
| tRNA(Ser) | 6012 | 6073 | | 62 | |  | | | |  | | |  | | | | | 220 | | | | | + | | | | | | |  | |
| tRNA(Glu) | 6294 | 6365 | | 72 | |  | | | |  | | |  | | | | | 220 | | | | | + | | | | | | |  | |
| tRNA(Phe) | 6586 | 6654 | | 69 | |  | | | |  | | |  | | | | | 2 | | | | | - | | | | | | |  | |
| ND5 | 6657 | 8324 | | 1668 | | 555 | | | | ATA | | | TAA | | | | | 0 | | | | | - | | | | | | |  | |
| tRNA(His) | 8325 | 8388 | | 64 | |  | | | |  | | |  | | | | | 18 | | | | | - | | | | | | |  | |
| ND4 | 8407 | 9738 | | 1332 | | 443 | | | | ATG | | | TAA | | | | | 46 | | | | | - | | | | | | |  | |
| ND4L | 9785 | 10069 | | 285 | | 94 | | | | ATA | | | TAG | | | | | 244 | | | | | - | | | | | | |  | |
| tRNA(Thr) | 10314 | 10383 | | 70 | |  | | | |  | | |  | | | | | 105 | | | | | + | | | | | | |  | |
| tRNA(Pro) | 10489 | 10554 | | 66 | |  | | | |  | | |  | | | | | 79 | | | | | - | | | | | | |  | |
| ND6 | 10634 | 11167 | | 534 | | 177 | | | | ATG | | | TAA | | | | | 39 | | | | | + | | | | | | |  | |
| CYTB | 11207 | 12325 | | 1119 | | 372 | | | | ATG | | | TAA | | | | | 27 | | | | | + | | | | | | |  | |
| tRNA(Ser) | 12353 | 12421 | | 69 | |  | | | |  | | |  | | | | | 80 | | | | | + | | | | | | |  | |
| ND1 | 12502 | 13446 | | 945 | | 314 | | | | ATT | | | TAA | | | | | 0 | | | | | - | | | | | | |  | |
| tRNA(Leu) | 13447 | 13518 | | 72 | |  | | | |  | | |  | | | | | 0 | | | | | - | | | | | | |  | |
| 16S rRNA | 13519 | 14877 | | 1359 | |  | | | |  | | |  | | | | | 0 | | | | | - | | | | | | |  | |
| tRNA(Val) | 14878 | 14952 | | 75 | |  | | | |  | | |  | | | | | 40 | | | | | - | | | | | | |  | |
| 12S rRNA | 14993 | 15774 | | 782 | |  | | | |  | | |  | | | | | 566 | | | | | - | | | | | | |  | |
| tRNA(Met) | 16341 | 16413 | | 73 | |  | | | |  | | |  | | | | | 12 | | | | | + | | | | | | |  | |
| tRNA(Ile) | 16426 | 16492 | | 67 | |  | | | |  | | |  | | | | | 159 | | | | | + | | | | | | |  | |
| tRNA(Gln) | 16652 | 16720 | | 69 | |  | | | |  | | |  | | | | | 68 | | | | | - | | | | | | |  | |
| ND2 | 16789 | 17757 | | 969 | | 322 | | | | ATA | | | TAA | | | | | 3 | | | | | + | | | | | | |  | |
| tRNA(Trp) | 17761 | 17829 | | 69 | |  | | | |  | | |  | | | | | 139 | | | | | + | | | | | | |  | |
| tRNA(Cys) | 17969 | 18034 | | 66 | |  | | | |  | | |  | | | | | 102 | | | | | - | | | | | | |  | |
| tRNA(Tyr) | 18137 | 18205 | | 69 | |  | | | |  | | |  | | | | | 205 | | | | | - | | | | | | |  | |
| 13 |  |  | | ***Tetraponera  aethiops*** | | | | | | | |  | | | |  | | | | |  | | | | |  | | |  | | |
| **Gene** | **Position** | | | **Size** | | | | **Codon** | | | | | | | | | **Intergenic** | | | | |  | | | | |  | | | |  |
|  | **From** | **To** | | **Nucleotide** | | **Aminoacid** | | **Start** | | | | **Stop** | | | **nucleotide** | | | | |  | | | | |  | | |  | | | |
| COX1 | 1 | 1533 | | 1533 | | 510 | | ATG | | | | TAA | | | 22 | | | | | + | | | | |  | | |  | | | |
| tRNA(Leu) | 1556 | 1625 | | 70 | |  | |  | | | |  | | | 0 | | | | | + | | | | |  | | |  | | | |
| COX2 | 1626 | 2300 | | 675 | | 224 | | ATT | | | | TAG | | | 3 | | | | | + | | | | |  | | |  | | | |
| tRNA(Lys) | 2304 | 2373 | | 70 | |  | |  | | | |  | | | 0 | | | | | + | | | | |  | | |  | | | |
| tRNA(Asp) | 2374 | 2440 | | 67 | |  | |  | | | |  | | | 0 | | | | | + | | | | |  | | |  | | | |
| ATP8 | 2441 | 2614 | | 174 | | 57 | | ATC | | | | TAA | | | 153 | | | | | + | | | | |  | | |  | | | |
| ATP6 | 2768 | 3436 | | 669 | | 222 | | ATG | | | | TAA | | | 25 | | | | | + | | | | |  | | |  | | | |
| COX3 | 3462 | 4241 | | 780 | | 259 | | ATG | | | | TAA | | | 7 | | | | | + | | | | |  | | |  | | | |
| tRNA(Gly) | 4249 | 4314 | | 66 | |  | |  | | | |  | | | 144 | | | | | + | | | | |  | | |  | | | |
| ND3 | 4459 | 4806 | | 348 | | 115 | | ATT | | | | TAA | | | 57 | | | | | + | | | | |  | | |  | | | |
| tRNA(Ala) | 4864 | 4934 | | 71 | |  | |  | | | |  | | | 1 | | | | | + | | | | |  | | |  | | | |
| tRNA(Arg) | 4936 | 5001 | | 66 | |  | |  | | | |  | | | 5 | | | | | + | | | | |  | | |  | | | |
| tRNA(Asn) | 5007 | 5074 | | 68 | |  | |  | | | |  | | | 1 | | | | | + | | | | |  | | |  | | | |
| tRNA(Ser) | 5076 | 5135 | | 60 | |  | |  | | | |  | | | 16 | | | | | + | | | | |  | | |  | | | |
| tRNA(Glu) | 5152 | 5223 | | 72 | |  | |  | | | |  | | | 8 | | | | | + | | | | |  | | |  | | | |
| tRNA(Phe) | 5232 | 5298 | | 67 | |  | |  | | | |  | | | 0 | | | | | - | | | | |  | | |  | | | |
| ND5 | 5299 | 6979 | | 1681 | | 560 | | ATT | | | | T- | | | 0 | | | | | - | | | | |  | | |  | | | |
| tRNA(His) | 6980 | 7047 | | 68 | |  | |  | | | |  | | | 11 | | | | | - | | | | |  | | |  | | | |
| ND4 | 7059 | 8384 | | 1326 | | 441 | | ATG | | | | TAG | | | 0 | | | | | - | | | | |  | | |  | | | |
| ND4L | 8385 | 8667 | | 283 | | 94 | | ATT | | | | T- | | | 2 | | | | | - | | | | |  | | |  | | | |
| tRNA(Thr) | 8670 | 8740 | | 71 | |  | |  | | | |  | | | 7 | | | | | + | | | | |  | | |  | | | |
| tRNA(Pro) | 8748 | 8818 | | 71 | |  | |  | | | |  | | | 31 | | | | | - | | | | |  | | |  | | | |
| ND6 | 8850 | 9380 | | 531 | | 176 | | ATA | | | | TAA | | | 38 | | | | | + | | | | |  | | |  | | | |
| CYTB | 9419 | 10531 | | 1113 | | 370 | | ATG | | | | TAA | | | 43 | | | | | + | | | | |  | | |  | | | |
| tRNA(Ser) | 10575 | 10643 | | 69 | |  | |  | | | |  | | | 10 | | | | | + | | | | |  | | |  | | | |
| ND1 | 10654 | 11604 | | 951 | | 316 | |  | | | | TAA | | | 0 | | | | | - | | | | |  | | |  | | | |
| tRNA(Leu) | 11605 | 11671 | | 67 | |  | |  | | | |  | | | 13 | | | | | - | | | | |  | | |  | | | |
| 16S rRNA | 11685 | 12979 | | 1295 | |  | |  | | | |  | | | 0 | | | | | - | | | | |  | | |  | | | |
| tRNA(Val) | 12980 | 13043 | | 64 | |  | |  | | | |  | | | 3 | | | | | - | | | | |  | | |  | | | |
| 12S rRNA | 13047 | 13811 | | 765 | |  | |  | | | |  | | | 548 | | | | | - | | | | |  | | |  | | | |
| tRNA(Met) | 14360 | 14429 | | 70 | |  | |  | | | |  | | | 9 | | | | | + | | | | |  | | |  | | | |
| tRNA(Ile) | 14439 | 14507 | | 69 | |  | |  | | | |  | | | 28 | | | | | + | | | | |  | | |  | | | |
| tRNA(Gln) | 14536 | 14605 | | 70 | |  | |  | | | |  | | | 58 | | | | | - | | | | |  | | |  | | | |
| ND2 | 14664 | 15629 | | 966 | | 321 | | ATA | | | | TAA | | | 37 | | | | | + | | | | |  | | |  | | | |
| tRNA(Trp) | 15667 | 15739 | | 73 | |  | |  | | | |  | | | -8 | | | | | + | | | | |  | | |  | | | |
| tRNA(Cys) | 15732 | 15801 | | 70 | |  | |  | | | |  | | | 73 | | | | | - | | | | |  | | |  | | | |
| tRNA(Tyr) | 15875 | 15941 | | 67 | |  | |  | | | |  | | | 47 | | | | | - | | | | |  | | |  | | | |
| 14 |  |  | | ***Tetraponera  rufonigra*** | | | | | | | |  | | | |  | | | | |  | | | | |  | | |  | | |
| **Gene** | **Position** | | | **Size** | | | | **Codon** | | | | | | | | | **Intergenic** | | | | |  | | | | |  | | | |  |
|  | **From** | **To** | | **Nucleotide** | | **Aminoacid** | | **Start** | | | | **Stop** | | | **nucleotide** | | | | |  | | | | |  | | |  | | | |
| COX1 | 1 | 1533 | | 1533 | | 510 | | ATG | | | | TAA | | | 14 | | | | | + | | | | |  | | |  | | | |
| tRNA(Leu) | 1548 | 1615 | | 68 | |  | |  | | | |  | | | 0 | | | | | + | | | | |  | | |  | | | |
| COX2 | 1616 | 2290 | | 675 | | 224 | | ATT | | | | TAA | | | 3 | | | | | + | | | | |  | | |  | | | |
| tRNA(Lys) | 2294 | 2362 | | 69 | |  | |  | | | |  | | | 0 | | | | | + | | | | |  | | |  | | | |
| tRNA(Asp) | 2363 | 2432 | | 70 | |  | |  | | | |  | | | 0 | | | | | + | | | | |  | | |  | | | |
| ATP8 | 2433 | 2606 | | 174 | | 57 | | ATC | | | | TAA | | | 149 | | | | | + | | | | |  | | |  | | | |
| ATP6 | 2756 | 3424 | | 669 | | 222 | | ATG | | | | TAA | | | 21 | | | | | + | | | | |  | | |  | | | |
| COX3 | 3446 | 4225 | | 780 | | 259 | | ATG | | | | TAA | | | 13 | | | | | + | | | | |  | | |  | | | |
| tRNA(Gly) | 4239 | 4308 | | 70 | |  | |  | | | |  | | | 162 | | | | | + | | | | |  | | |  | | | |
| ND3 | 4471 | 4818 | | 348 | | 115 | | ATT | | | | TAA | | | 33 | | | | | + | | | | |  | | |  | | | |
| tRNA(Ala) | 4852 | 4916 | | 65 | |  | |  | | | |  | | | 1 | | | | | + | | | | |  | | |  | | | |
| tRNA(Arg) | 4918 | 4981 | | 64 | |  | |  | | | |  | | | 3 | | | | | + | | | | |  | | |  | | | |
| tRNA(Asn) | 4985 | 5051 | | 67 | |  | |  | | | |  | | | 1 | | | | | + | | | | |  | | |  | | | |
| tRNA(Ser) | 5053 | 5109 | | 57 | |  | |  | | | |  | | | 13 | | | | | + | | | | |  | | |  | | | |
| tRNA(Glu) | 5123 | 5193 | | 71 | |  | |  | | | |  | | | 8 | | | | | + | | | | |  | | |  | | | |
| tRNA(Phe) | 5202 | 5267 | | 66 | |  | |  | | | |  | | | 0 | | | | | - | | | | |  | | |  | | | |
| ND5 | 5268 | 6942 | | 1675 | | 558 | | ATT | | | | T- | | | 0 | | | | | - | | | | |  | | |  | | | |
| tRNA(His) | 6943 | 7012 | | 70 | |  | |  | | | |  | | | 22 | | | | | - | | | | |  | | |  | | | |
| ND4 | 7035 | 8354 | | 1320 | | 439 | | ATG | | | | TAG | | | 0 | | | | | - | | | | |  | | |  | | | |
| ND4L | 8355 | 8637 | | 283 | | 94 | | ATT | | | | T- | | | 2 | | | | | - | | | | |  | | |  | | | |
| tRNA(Thr) | 8640 | 8711 | | 72 | |  | |  | | | |  | | | 7 | | | | | + | | | | |  | | |  | | | |
| tRNA(Pro) | 8719 | 8788 | | 70 | |  | |  | | | |  | | | 46 | | | | | - | | | | |  | | |  | | | |
| ND6 | 8835 | 9359 | | 525 | | 174 | | ATG | | | | TAA | | | 23 | | | | | + | | | | |  | | |  | | | |
| CYTB | 9383 | 10495 | | 1113 | | 370 | | ATG | | | | TAA | | | 17 | | | | | + | | | | |  | | |  | | | |
| tRNA(Ser) | 10513 | 10583 | | 71 | |  | |  | | | |  | | | 31 | | | | | + | | | | |  | | |  | | | |
| ND1 | 10615 | 11565 | | 951 | | 316 | | ATT | | | | TAG | | | 0 | | | | | - | | | | |  | | |  | | | |
| tRNA(Leu) | 11566 | 11631 | | 66 | |  | |  | | | |  | | | 0 | | | | | - | | | | |  | | |  | | | |
| 16S rRNA | 11632 | 12915 | | 1284 | |  | |  | | | |  | | | 0 | | | | | - | | | | |  | | |  | | | |
| tRNA(Val) | 12916 | 12980 | | 65 | |  | |  | | | |  | | | 3 | | | | | - | | | | |  | | |  | | | |
| 12S rRNA | 12984 | 13744 | | 761 | |  | |  | | | |  | | | 568 | | | | | - | | | | |  | | |  | | | |
| tRNA(Met) | 14313 | 14380 | | 68 | |  | |  | | | |  | | | 14 | | | | | + | | | | |  | | |  | | | |
| tRNA(Ile) | 14395 | 14463 | | 69 | |  | |  | | | |  | | | 43 | | | | | + | | | | |  | | |  | | | |
| tRNA(Gln) | 14507 | 14575 | | 69 | |  | |  | | | |  | | | 58 | | | | | - | | | | |  | | |  | | | |
| ND2 | 14634 | 15599 | | 966 | | 321 | | ATA | | | | TAA | | | 18 | | | | | + | | | | |  | | |  | | | |
| tRNA(Trp) | 15618 | 15685 | | 68 | |  | |  | | | |  | | | -8 | | | | | + | | | | |  | | |  | | | |
| tRNA(Cys) | 15678 | 15740 | | 63 | |  | |  | | | |  | | | 78 | | | | | - | | | | |  | | |  | | | |
| tRNA(Tyr) | 15819 | 15883 | | 65 | |  | |  | | | |  | | | 24 | | | | | - | | | | |  | | |  | | | |
