# Supplementary material for: Accessible molecular phylogenomics at no cost: obtaining 14 new mitogenomes for the ant subfamily Pseudomyrmecinae from public data
Source: PeerJ. 2019 Jan 24;7:e6271. doi: 10.7717/peerj.6271 (PMC6348091; doi:10.7717/peerj.6271)
Supplement: Table S3 [file peerj-07-6271-s005.docx]

| Accession Number | Species name | Reference |
| --- | --- | --- |
| NC_028534 | *Linepithema humile* | Bi et al., Unpublished |
| NC_023093 | *Leptomyrmex pallens* | Berman, Austin & Miller, 2014 |
| MF417380 | *Atta texana* | Almeida,C.S., Unpublished |
| NC_026133 | *Myrmica scabrinodis* | Babbucci et al., 2014 |
| KX951753 | *Cardiocondyla obscurior* | Liu & Qian, Unpublished |
| NC_015075 | *Pristomyrmex punctatus* | Hasegawa et al., 2011 |
| NC_030541 | *Wasmannia auropunctata* | Duan, Peng & Qian, 2016 |
| NC_030176 | *Vollenhovia emeryi* | Liu et al., 2016 |
| NC_014669 | *Solenopsis geminata* | Gotzek, Clarke & Shoemaker, 2010 |
| NC_014672 | *Solenopsis invicta* | Gotzek, Clarke & Shoemaker, 2010 |
| NC_014677 | *Solenopsis richteri* | Gotzek, Clarke & Shoemaker, 2010 |
| NC_029357 | *Camponotus atrox* | Berman, Austin & Miller, 2014 |
| NC_030790 | *Polyrhachis dives* | Song et al., Unpublished |
| NC_026132 | *Formica fusca* | Babbucci et al., 2014 |
| NC_026711 | *Formica selysi* | Yang et al., 2016 |
| NC_001566 | *Apis mellifera ligustica* | Crozier & Crozier, 1993 |
| NC_010967 | *Bombus ignitus* | Cha et al., 2007 |
| *BK010475* | *P. concolor* | This work |
| *BK010473* | *P. dendroicus* | This work |
| *BK010474* | *P. elongatus* | This work |
| *BK010379* | *P. feralis* | This work |
| *BK010380* | *P. ferrugineus* | This work |
| *BK010381* | *P. flavicornis* | This work |
| *BK010472* | *P. gracilis* | This work |
| *BK010382* | *P. janzeni* | This work |
| *BK010383* | *P. pallidus* | This work |
| *BK010384* | *P. particeps* | This work |
| *BK010385* | *P. peperi* | This work |
| *BK010386* | *P. veneficus* | This work |
| *BK010476* | *T. aethiops* | This work |
| BK010387 | *T. rufonigra* | This work |
